# Leveraging feature selection for enhanced fall risk prediction in elderly using gait analysis

**DOI:** 10.1007/s11517-024-03180-2

**Published:** 2024-08-10

**Authors:** Sabri Altunkaya

**Affiliations:** Department of Electrical and Electronics Engineering, Necmettin Erbakan University, Konya, Türkiye

**Keywords:** Gait measures, Accelerometer, Fall prediction, Fall risk

## Abstract

There is no effective fall risk screening tool for the elderly that can be integrated into clinical practice. Developing a system that can be easily used in primary care services is a current need. Current studies focus on the use of multiple sensors or activities to achieve higher accuracy. However, multiple sensors and activities reduce the availability of these systems. This study aims to develop a system to perform fall prediction for the elderly by using signals recorded from a single sensor during a short-term activity. A total of 168 features in the time and frequency domains were created using acceleration signals obtained from 71 elderly people. The features were weighted based on the ReliefF algorithm, and the artificial neural networks model was developed using the most important features. The best classification result was obtained using the 17 most important features of those weighted for K = 20 nearest neighbors. The highest accuracy was 82.2% (82.9% Sensitivity, 81.6% Specificity). The partially high accuracy obtained in our study shows that falling can be detected early with a sensor and a simple activity by determining the right features and can be easily applied in the assessment of the elderly during routine follow-ups.

**Graphical Abstract:**

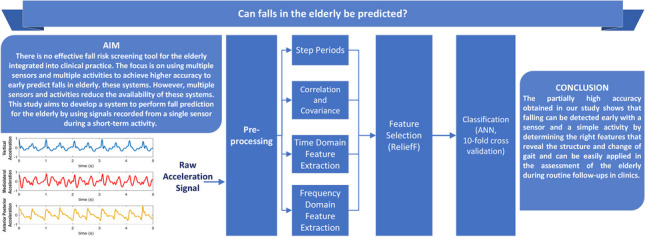

**Supplementary Information:**

The online version contains supplementary material available at 10.1007/s11517-024-03180-2.

## Introduction

Balance is simply defined as the ability to maintain the body's center of gravity on the base of support. Maintaining an upright posture and balanced movement requires complex interplay between the nervous system and muscles. This system gathers information about the body’s position and movement through various senses (proprioception, vision, and vestibular). This sensory data is then processed by the central nervous system, and the processed information is used to send coordinated signals to the muscles, allowing them to adjust posture [1]. Any disruption in the above- mentioned senses or in the muscles that process the response created because of the senses may cause a fall. With aging, sensory acuity and motor skills decrease [1, 2], and falls may occur in the elderly due to aging [3–6]. Falling in the elderly is a public health problem that usually requires hospital care and may result in the death of the patient in some cases. Death occurs in 20% of patients when a hip fracture has occurred in a falling case [3, 7–9]. The probability of encountering these problems increases with increasing age [3, 10, 11]. Apart from the physical problems faced by the patient, some psychological issues such as movement restriction caused by fear of falling again, withdrawal from social life, and depression occur during the postoperative period [9]. It is known that 25 million dollars are spent for the treatment of fall-related injuries per year in the European Union [12], while 50 million dollars are spent in the USA [13]. It is reported that with increased life expectancy, the elderly population will increase exponentially in the next 10 to 20 years [7, 9, 10, 12]. With the enhanced population of the elderly in the world, falling-related problems and expenses will increase [8, 9, 12, 13]. Prevention of falls in the elderly, whose physical, psychological, and financial effects are quite serious, is very important for both society and individuals [9, 14–16]. In order to prevent falls, the American Geriatrics Society and the British Geriatrics Society recommend that all adults over the age of 65 be screened annually for a history of falls or balance disorders. In this assessment, according to the American Geriatrics Society/British Geriatrics Society (AGS/BGS) clinical practice guideline, answers to questions about falls ("Fell in last year? Feels unsteady when standing or walking?, Worries about falling?") are obtained. According to the answer, the elderly person's gait, strength, and balance are evaluated using TUG test and/or 30-Second Chair Stand and 4-Stage Balance test. Depending on the test result and the number of falls, the need for further evaluation is determined or the necessary recommendations are made [15, 17, 18]. There are certain problems such as those questionnaires and simple physiological tests used for fall risk assessment are subjective and qualitative, rather than being a precise and objective method [19–21], while TUG is the most widely used test, despite its low level of prediction validity [22–24]. Computer-controlled advanced devices using multiple sensors are difficult to use in primary care services due to disadvantages such as space, a requirement for a specialist, test duration, and cost [20, 25]. Accordingly, effective fall risk screening is still not routinely integrated into clinical practice [26]. Therefore, it has been concluded that an inexpensive, easy-to-use, objective and quantitative method is necessary for the early detection and prevention of falls in the elderly for both the elderly to live more comfortably and to reduce the cost of social insurance [2, 20, 27].

We can classify the studies performed for fall prediction in terms of the type of sensor used, the number of sensors, and the physical activity performed during recording. Sensors such as accelerometers, force-sensitive platforms, pressure-sensing insoles, and depth cameras have been used for the early detection of fall incidents [6, 26, 28]. Accelerometers have recently become widespread since they are easy to use and financially advantageous in assessing the risk of falling [2, 3, 20, 29–31]. Accelerometers are placed in different positions such as the head, upper back, breastbone, shoulder, elbow, wrist, hip, waist, thigh, knee, ankle, and foot. The most used position among these is the waist [3, 20, 25]. The TUG test, the Sit to Stand test, the Alternate Step Test, the Standing Postural Sway, and walking for a certain distance or duration are among the exercises that are carried out [22–24].

Chen et al. obtained the time–frequency definitions of the acceleration signals recorded from the accelerometer placed at vertebrae L3–L5 during the TUG test using wavelet transformation, and they obtained 94.1% accuracy by using the time–frequency definitions in the training and testing of the Stacked Autoencoder Network Architecture [32]. Qui et al. extracted gait-based features from acceleration signals recorded from five different acceleration sensors during five different tests. Later, they classified these features using support vector machines (SVM) and achieved a classification accuracy of 89.4% [33]. Rivolta et al. used acceleration signals recorded from an accelerometer placed on the chest during the Tineeti test and achieved 89% accuracy with ANN [7]. Gietzelt et al. extracted the features obtained from one-week walking signals of elderly people with dementia and classified them using a decision trees algorithm and achieved 88.5% accuracy [34]. Grene et al. extracted the features obtained from the signals recorded from five different acceleration sensors during three different activities and achieved an accuracy of 87.58% for men and 78.11% for women using SVM [35]. Howcroft et al. achieved 84% accuracy by classifying the features obtained from the signals recorded from pressure-sensing insoles and head, pelvis, and left shank accelerometers with a multi-layer perceptron neural network [28]. Yu et al. evaluated the features obtained from the acceleration signals recorded during the TUG test and some demographic information using the short form Berg balance scale and achieved 84% accuracy [11]. Apart from the best results we summarized above, similar studies for multisensor or activity with accuracy varying between 84 and 57% can be seen in the literature [5, 12, 30, 36–40].

The highest accuracy is 84% in those evaluating accelerometer data recorded from an accelerometer during walking at a normal pace [12, 24, 28, 37, 41, 42]. Howcroft et al. achieved 84% accuracy and 66.7% sensitivity when they classified the features obtained from the signals recorded from an accelerometer placed on the head with SVM [28]. Busiret et al. divided the accelerometer and gyrometer signals recorded during a 6-min walk into 10-s windows and used them as input in a CNN-based deep learning model. They achieved 81% accuracy and 62.5% sensitivity in the model they created [12]. In another study published by Howcroft et al. in 2017, they classified the features obtained from acceleration signals recorded over the pelvis using artificial neural networks and obtained 77.8% and 71.4% accuracy and sensitivity, respectively [37]. The common point of the above three studies is their low sensitivity. The low sensitivity indicates that these three studies had difficulty finding fallers. In their study conducted with only female participants, Hua et al. obtained an accuracy of 78.9% and a sensitivity value of 87.7% when they classified gait-based features obtained from acceleration signals using the random forest algorithm [41]. Although it has the best sensitivity value among studies conducted with one sensor and one activity, the fact that the participants were only women limits the generalizability of the study. In addition, in Greene’s study [35], which gave separate results for men and women, it was seen that female participants showed higher sensitivity. It may not be correct to compare Hua’s single-sex study with mixed-sex studies. Nait et al. evaluated raw acceleration signals using different deep learning and multitask learning algorithms. For this purpose, they divided the acceleration signals into 10-s intervals and made sample-based and subject-based classifications. The study results were evaluated with receiver operating characteristic (ROC) curves, and 0.75 AUC was obtained in subject-based classification [24]. In both Nait et al. and Busiret et al. studies [12, 24], raw acceleration signals were directly used as input in a deep learning-based algorithm, but the desired high success was not achieved in either study. Ihlen et al. attempted to detect fallers using a partial least squares regression model using a combination of phase-dependent generalized multiscale entropy, gait, and demographic-based features [42]. They achieved 83% accuracy and 83% sensitivity, which is the best result among single-sensor, single-activity studies. The difference between Ihlen’s study and other studies is that it uses features obtained in different ways in addition to standard gait analysis-based features. It can be seen that determining the correct features, which is a point we focus on in our study, can increase the classification accuracy. For this purpose, in our study, unlike existing studies, in addition to basic gait attributes, correlation covariance-based, time domain, frequency domain, and related statistical features were determined to create a wide feature pool, and the most effective ones were selected among them.

As can be understood from the literature, most studies have focused on solutions such as performing different activities, placing more sensors on the body, or placing an accelerometer in multiple parts of the body to obtain better results. However, this solution is not applicable quickly and easily due to a large number of sensor groups and the difficulty of performing different activities. In the studies on one sensor and one activity in the literature, sufficient accuracy and sensitivity could not be achieved because a limited number of features were used. In this study, we aimed to develop a model that can distinguish the elderly with a high probability of falling by using acceleration signals recorded during a walking activity at a normal pace for a short distance based on the accelerometer placed on the waist.

## Materials and methods

### Database

In this study, the long-term movement monitoring (LTMM) database in PhysioNet was used [43, 44]. The LTMM database consists of three-day and one-minute acceleration signals recorded from 71 elderly people for fall risk, balance, and gait studies. The average age of the participants was 78.36 ± 4.71, ranging between 65 and 87. Three-day recordings in the LTMM database was taken through participants' own use of the accelerometer belt in a home environment. The one-minute recording in the LTMM database was taken during the participants' one-minute walk in a straight line at their own chosen pace, wearing an accelerometer belt in a laboratory environment. Depending on participants' reports about their previous falls, Weiss et al. classified 38 people as non-fallers and 35 as fallers [44]. If the elderly had fallen twice in the past 12 months, they were included in the faller group, and the others were included in the non-fallers group. Detailed information about the recording device and recording protocol can be found in [44]. This study aims to develop a simple method for use in primary care services. Therefore, it has been concluded that one-minute recordings in the database will be more accurate for the purposes of the current study in terms of obtaining them in a short time. Thus, one-minute accelerometer recordings in the LTMM database were used in the current study.

### Preprocessing

**Fig. 1:**
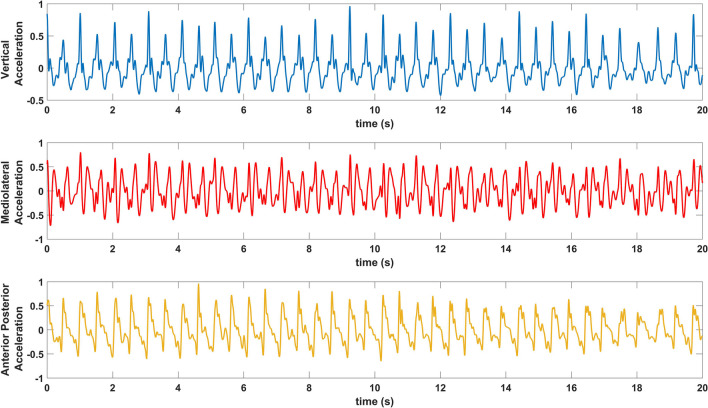
A sample of 3 axes pre-processed acceleration signal

**Fig. 2:**
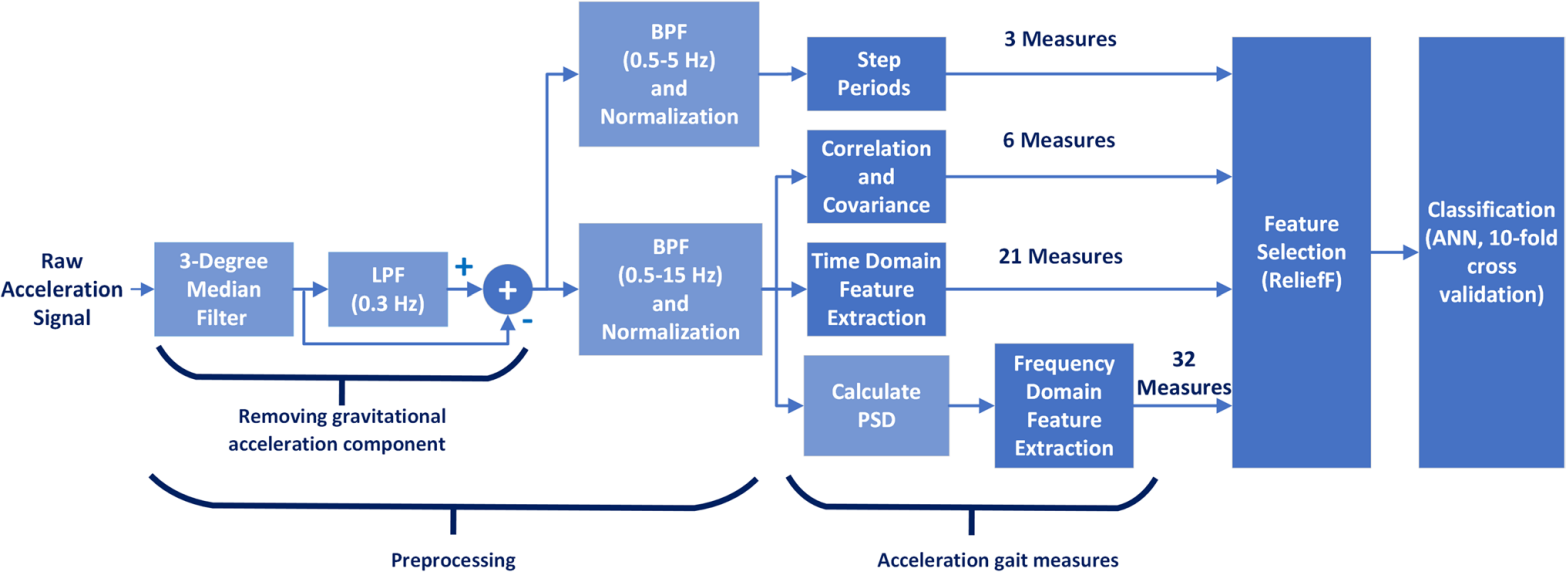
Workflow of preprocessing, feature extraction, feature selection and classification of this study

All three-accelerometer axes (Vertical (V), Mediolateral (ML), and Anterior–Posterior (AP)) signals have been first filtered with a 3-degree median filter to reduce the effect of sudden changes, and then the gravitational acceleration component has been removed to obtain body acceleration [45]. Then two different filtering processes were applied. First, obtained body acceleration signals are passed through a 0.5 Hz-3 Hz bandpass filter to determine step periods [46]. Second, since 99% of the walking signal energy is below the 15 Hz band, the body acceleration signals are passed through a 0.5 Hz-15 Hz bandpass filter for use in analyses [47]. Both signals have then been normalized to the maximum. A sample of 0.5 Hz-15 Hz bandpass filtered and normalized acceleration signals of the three axes can be seen in Fig. 1. A flow diagram of the methods used in processing acceleration signals is shown in Fig. 2. These processes have also been applied to the acceleration signals of the three axes except calculation of step period, correlation, and covariance blocks.

### Acceleration gait measures

In gait analysis, measurements from the acceleration signal are generally performed in five categories. These include gait cycle event timings; statistical features; signal frequency features; time–frequency features; and information-theoretic features [48]. Gait cycle event timing, statistical features, and frequency spectrum features were used in this study. Three related to basic gait measurement (average step duration, average stride duration, cadence) were calculated for only the V accelerometer axes, six on the inter-acceleration axes correlation and covariance were calculated between V and ML axes, AP and ML axes, V and AP axes for both correlation and covariance, 21 time domain and 32 frequency domain features were calculated separately for each acceleration axis (Fig. 2). As a result, a total of 168 features belonging to a person were obtained, including the first nine features calculated only once for a person and, 53 features calculated separately for the three acceleration axes recorded from a person. The statistical features have been calculated for both the acceleration signal time series and the acceleration signal frequency spectrum. The feature pool has been expanded through distinguishing measurement of certain statistical features for the first and the last half of steps for time series, distinguishing measurement of certain statistical features for different bands for frequency spectrum, and measurement of features such as Centroid, Spread, Decrease, Flatness and Crest used in audio signals for the frequency spectrum. While calculating the time-domain features, the calculation has been performed for each step of each participant, and the average has been taken. Local maximums of the vertical axis acceleration signal were used to determine the steps [5, 49]. The power spectral density (PSD) of acceleration signals was estimated using the periodogram method with a Tukey window of cosine fraction of 0.5. A detailed description of the measures is provided in the supplementary material.

### Feature selection

ReliefF is a heuristic estimator of feature quality effectively used for datasets consisting of conditionally dependent and independent features, in addition to noisy features [50]. This algorithm functions by assigning a reward to features that give different values to nearest neighbors (K) of different classes, while assigning a punitive to features that give different values to nearest neighbors (K) of the same class. Accordingly, it calculates a weight for each feature and determines how much that feature will help to distinguish the class [51]. The ReliefF was used in the current study since it has been previously found to be the feature selection algorithm that provides the best sensitivity [52]. By changing the number of nearest neighbors from K = 1 to K = 40, the ReliefF algorithm has been run 40 times, and 40 differently weighted feature sets have been obtained. The number of features to be used after weighting has been determined experimentally. For this purpose, different feature sets are created by increasing the number of features one by one, from 5 to 40 features with the highest weight. For each obtained feature set, the classification process is repeated to find the feature set with the highest accuracy [52].

### Classification

Multilayer perceptron (MLP) structures are considered an important class of artificial neural networks (ANN). MLP consists of an input layer, a hidden layers, and an output layer, and is trained with the back-propagation algorithm [53, 54]. The current study uses an MLP trained with a back-propagation algorithm to classify fallers and non-fallers. The MLP structure and training have been carried out with Orange Data Mining, which is a machine-learning software package used extensively in data mining for scientific studies [55]. Rectified linear unit function (RELU) as the activation function of the hidden and output layers and stochastic gradient-based optimizer as the solver for the weight optimization were chosen in Orange Data Mining. The regularization term (alpha) is 0.0001 and the maximum number of iterations is 200. Other parameters have been set to default based on the Scikit-learn module [56]. The number of neurons in the input layer is provided by the number of selected input features. The output layer consists of one layer indicating the fallers and non-fallers. The number of neurons in hidden layers has been changed, starting from 10 by 10 increments up to 200, and the best accuracy has been aimed to be achieved.

## Results

A total of 168 features have been calculated for each record, including three for the gait cycle; 6 for the inter-axis correlation and covariance between axes, and 21 time and 32 frequency domain features calculated separately for the three axes. When the feature selection process is applied using the Relieff algorithm, 82.2% accuracy (82.9% sensitivity and 81.6% specificity) is obtained as the best classification result with 17 of the most important weighted features for the nearest neighbor number K = 20. The best classification result was obtained using 50 neurons in hidden layers in the ANN structure. Close classification accuracies have also been obtained from different weighted numbers of features for the nearest neighbor numbers K = 20, 21, and 23 (Table 2). However, since the number of features used to obtain these results is greater than 17 and the sensitivity is lower, it has been decided that the best classification result belongs to the 17 features obtained by searching for K = 20 nearest neighbors. The names of the features for which the highest accuracy has been obtained, and the average and standard deviation of the features of the groups are given in Table 1. The classification results obtained with different numbers of features and nearest neighbors are given in Table 2.

**Table 1:** Names of the 17 features that give the highest accuracy, the domain and the acceleration axis from which the features are obtained, and the mean and standard deviation of the features for each group

Weight Order	Feature Name	Features Domain	Acceleration Axis	Non-Fallers		Fallers	
				Mean	Std	Mean	Std
1	StpReg	Time	V	0.650	0.191	0.431	0.227
2	kurt_PSD	Frequency	ML	0.250	0.252	0.197	0.227
3	corr_ML_AP	Time	K	0.497	0.236	0.476	0.245
4	wF2	Frequency	AP	0.241	0.207	0.297	0.237
5	StrReg	Time	V	0.530	0.237	0.389	0.161
6	flatness_PSD	Frequency	V	0.154	0.167	0.192	0.284
7	skew_PSD	Frequency	ML	0.400	0.239	0.429	0.192
8	mean_LH_PSD	Frequency	V	0.066	0.102	0.115	0.238
9	med_PSD	Frequency	V	0.071	0.098	0.114	0.233
10	pF2	Frequency	V	0.127	0.110	0.170	0.091
11	AmpF2	Frequency	V	0.177	0.160	0.270	0.219
12	med_PSD	Frequency	ML	0.116	0.208	0.068	0.137
13	cov_ML_AP	Time	K	0.362	0.181	0.350	0.126
14	mean_LH_PSD	Frequency	ML	0.114	0.208	0.064	0.136
15	meanTrend	Time	V	0.509	0.222	0.472	0.148
16	crest_PSD	Frequency	AP	0.435	0.239	0.605	0.223
17	flatness_PSD	Frequency	ML	0.152	0.207	0.089	0.116

AmpF2: Amplitude of the second dominant frequency, corr_ML_AP: Correlation between ML and AP axes acceleration, cov_ML_AP: Covariance between ML and AP axes acceleration, crest_PSD: Spectral crest, flatness_PSD: Spectral flatness, kurt_PSD: Spectral kurtosis, mean_LH_PSD: Mean of the last half of the PSD, meanTrend: Mean trend of the acceleration, med_PSD: Median of the PSD, pF2: Prominence of the second dominant peak, skew_PSD: Spectral skewness, StpReg: Step regularity, StrReg: Stride regularity, wF2: Width of the second dominant frequency

**Table 2:** Classification results for different numbers of features and nearest neighbor

K	N	AUC	Accuracy	Sensitivity	Specificity	TP	FN	FP	TN
20	17	0.863	0.822	0.829	0.816	29	6	7	31
23	28	0.840	0.822	0.800	0.842	28	7	6	32
20	31	0.874	0.822	0.771	0.868	27	8	5	33
23	31	0.881	0.822	0.771	0.868	27	8	5	33
21	35	0.851	0.822	0.771	0.868	27	8	5	33
21	36	0.851	0.822	0.800	0.842	28	7	6	32

**K**: The nearest neighbors used in the ReliefF algorithm, **N**: Number of features, **AUC**: Area under the curve, **TP**: True positive, **FN**: False negative, **FP**: False Positive, **TN**: True negative

## Discussion

The main purpose of this study is to perform early detection of falling, which is easy to use, inexpensive, does not require specialists, and does not disturb the patient. Therefore, for this purpose, it should be paid attention to use a small number of inexpensive sensors, place the sensor so as not to disturb the person, and select a short-term and easily applicable activity. In this context, when the literature is examined, previous studies can be classified according to the combination of sensors, the body part where the sensors are placed during recording, and the activity performed during recording.

Sensors such as accelerometers, force-sensitive platforms, pressure-sensing insoles, and depth cameras have been used in the early detection of falls [6, 26, 28]. In the literature, analyses have been performed based on the exclusive use of these sensors, the combination of different sensors, or the combination of the same sensor placed in different parts of the body [6]. In our study, acceleration signals obtained from accelerometers, which are the cheapest and easiest to use, have been used.

Regions such as the head, upper back, breastbone, shoulder, elbow, wrist, hip, waist, thigh, knee, ankle, and foot are preferred to place the sensors [3, 25]. Of these regions, the waist (the center of the body at the lower back), which has also been used in the current study, has been used in 65% of the studies. It is the most suitable region since it is the center of gravity of the body and allows the sensor to be placed with a basic belt without disturbing the patient [20]. In addition, in a study that has compared multiple sensors and multiple locations, it is seen that the waist is the region that provides the highest accuracy among single-sensor evaluations [37]. Therefore, it is considered that this layout is suitable for our study, which aims at simple use.

After the sensors are placed on the body, activities such as walking for a certain distance or duration, the TUG test, the Sit to Stand test, the Alternate Step Test, and the Standing Postural Sway are performed [20, 26, 57]. Walking at a normal pace is the most used activity in fall prevention studies [20, 29]. It is also considered the most appropriate activity in terms of being applicable in primary care services. Since the data set used in our study consists of the data collected during a 1-min walk at a normal pace, it provides an easy usage purpose and can be easily applied.

To our knowledge, in the literature, the highest accuracy is 94.1% among the studies conducted on the detection of falls in advance. This result has been obtained with deep learning classification of the time–frequency analysis images of the anteroposterior axis acceleration signal recorded during the TUG test of the elderly classified as fallers and non-fallers according to the TUG test score [32]. Similarly, 89% accuracy has been obtained by using the features obtained from the acceleration signals recorded during the Tinetti test from the elderly, who are divided into those with a high risk of falling according to the total Tinetti score and those who are not [7]. However, there is no acceptable predictive validity for any of the tests such as the TUG and Tinetti tests, which are recommended to be used exclusively in determining the fallers and non-fallers [22–24]. Accordingly, studies in which the data are grouped by the pre and/or post study fall history will be more subjective in determining the fallers. To our knowledge, the highest accuracy is 89.7% among the studies which determine the fallers and non-fallers according to the fall story. In this study, the feature set obtained from accelerations of the transition from sitting to standing was selected from the acceleration signal recorded in a three-day home environment, and the features obtained from laboratory functional measures have been used [38]. The closest accuracy to the above result is 89.4%, obtained by classifying 38 features from the acceleration signals recorded from 5 different sensors for seven different activities. However, it is stated in the study that the time taken for applying seven different activities with five sensors is 30 min [33]. In another study using five acceleration sensors, a pressure sensor, and a balance board, the attributes obtained from the acceleration signals recorded during the TUG test, five-times-sit-to-stand test, and balance evaluation have been classified. In this study, 87.58% accuracy was obtained for males and 78.11% for females [35]. In the literature, except for the above studies, those with an accuracy starting from 84% [28] and lower may be found for multiple sensors or multiple activities. However, the general problem related to multiple sensors and multiple activity studies is the length of data recording time and the need for excessive equipment.

The number of single-sensor single-activity studies is quite limited. Table 3 shows the studies performed with a single sensor and a single activity. Some of the studies were conducted with multiple sensors and multiple activities. The results of these studies with a single sensor and a single activity that achieved high accuracy have been taken and included in Table 3 [12, 28, 37]. In Table 3, two accuracies, two sensitivities, and two specificities are presented for the study given in the second line. One is the results obtained when both single and multiple falls have been included as the fallers in the data set, and the other is the results obtained when only multiple falls have been included as the fallers. Higher accuracy was obtained in the classification study in which multiple fallers are grouped as fallers [42]. When we compare this with the current study, it is seen that there are two studies with higher accuracy than ours with a slight difference. [28, 42]. Although one of the studies had an accuracy of 84%, its low sensitivity draws attention. Therefore, it can be concluded that it didn't correctly identify fallers compared to the current study [28]. In other study, with an accuracy rate of 83%, the classification process was performed based on walk records obtained from a one-week home recording, which had been first divided into epochs of 30 s, and then visually checked and classified [42]. In general, although there is no definite superiority among single-sensor single-activity studies, Ihlen's and the current study stand out in terms of accuracy and sensitivity.

**Table 3:** Summary of the studies conducted with a single sensor and single activity

	Study	Sensor & Location	Participants	Testing Tasks & Measures	Model	Validation Method	Accuracy (Acc) Sensitivity (Se) Specificity (Sp) in %
1	[28]^**a**^	Acc.& Head	76 Non-Fallers 24 Fallers	7.62 m walk & Temporal, DS, REOH, FFT Quartile, MLE	SVM	75% Train 25% Test	Acc = 84% Se = 66.7% Sp = 89.5%
2	[42]	Acc.& LB	199 Non-Fallers 58 Single Falls 46 Multiple Falls	30 s walking epoch & Entropy Measures	PLSR	80% Train 20% Test	Acc = 77%, 83% Se = 75%, 83% Sp = 80%,83%
3	[12]^**a**^	Acc.& LR	49 Non-Fallers 24 Fallers	6 min. walk & Raw Acc. signal	CNN	80% Train 20% Test	Acc = 81% Se = 62.5% Sp^b^
4	[41]	Acc.& Hip	47 Non-Fallers 19 Fallers (Only Woman)	400 m walk & Temporal, DS, cross-correlation	RF	tenfold cross- validation	Acc = 78.9% Se = 87.7% Sp^b^
5	[37]^**a**^	Acc.& Pelvis	47 Non-Fallers 28 Fallers	7.62 m walk with task & Temporal, DS, REOH, FFT Quartile, MLE	ANN	75% Train 25% Test	Acc = 77.8% Se = 71.4% Sp = 81.8%
6	[24]	Acc.& LB	195 Non-Fallers 101 Fallers	10-s walking epochs& Raw acc. signal	DNN CNN LSTM	tenfold cross- validation	Acc^b^ AUC = 0.63–0.96 Se^b^ Sp^b^
7	Our Study	Acc.& LB	38 Non-Fallers 35 Fallers	1 min walk& Time and Frequency domain	ANN	tenfold cross- validation	Acc = 82% Se = 80% Sp = 84.2% AUC = 0.847

Acc: Accelerometer, LB: Lower Back, LR: Lumbar Region, DS: Descriptive Statistics, REOH: Ratio of Even to Odd Harmonics, FFT: Fast Fourier Transform, MLE: Maximum Lyapunov Exponent. SVM: Support Vector Machine, PLSR: Partial Least Squares Regression, CNN: Convolutional Neural Network, RF: Random Forest, ANN: Artificial Neural Network, DNN: Deep Neural Network, LSTM: Long Short-Term Memory

^a^Part of the multi-sensor/activity study,

^b^There is no information

As a result of our study, our feature combination, which provided the best classification accuracy, consisted of five time-domain and 12 frequency-domain features. Of the features, 2 are obtained from the AP axis acceleration; 5 are obtained from the ML axis acceleration; 8 are obtained from V axis acceleration, and 2 are covariance and correlation between ML and AP axes accelerations.

Two of the time-domain features are vertical axis step and stride regularity, which are standard gait analysis features. In previous studies, it is seen that these two features have been used to distinguish classes [7] or to reveal the statistical difference between the groups [58]. Similar to the study conducted by [41], the correlation between the ML and AP axes acceleration is included in the features set that distinguish classes with the highest accuracy. Two time-domain features that we have not encountered before in the feature pool that separates the classes in the literature are included in our study. One is the covariance between the ML and AP axes acceleration, and the other is the vertical axis MeanTrend. The fact that the correlation between the ML and AP axes acceleration has been included in the feature pool both in the current study and in the literature has revealed its effectiveness in distinguishing groups similar to covariance, which is inversely related to correlation. The mean trend is a feature that is used in activity determination with accelerometers, and it provides effective results [59]. In this study, it is seen that the vertical axis MeanTrend is included in the feature combination that gives high accuracy in separating the fallers and non-fallers. Since the vertical axis acceleration signal is more variable in falling patients, [60], we conclude that the mean trend reflects the vertical axis variability.

In the current study, the frequency domain features included in the feature pool that provide the best classification accuracy are "wF2_PSD and crest_PSD" obtained from AP axis acceleration, "Kurt_PSD, Med_PSD, Mean_LH_PSD, flatness_PSD and skew_PSD" obtained from ML axis acceleration and “mean_LH_PSD, med_PSD, pF2, AmpF2 and flatness_PSD" obtained from V axis acceleration. Of these features, the standard frequency domain features such as the amplitude of peak frequency [44, 60], the width and relative prominence of the peaks [44, 60, 61], and the mean and median of the spectrum [62, 63] have been used in previous studies. The wF2_AP, mean_LH_PSD_ML, med_PSD_ML, AmpF2_V, pF2_V mean_LH_PSD_V, and med_PSD_V features, included in the feature pool in the current study, have been used in previous studies, and are reported to reveal a statistically significant difference between those the fallers and non-fallers. However, it has been concluded that these features are not used in classification-based studies. Other features that we obtain from the frequency domain (crest_PSD_AP, kurt_PSD_ML, skew_PSD_ML, flatness_PSD_ML, flatness_PSD_V), on the other hand, are not used both in studies examining whether there is a statistically significant difference between features and in those classifying groups through features. A higher risk for falls is associated with walking slower, less regular, less symmetric, and less stable, in addition to walking more variable and less smooth in VT and AP, while it is less variable, smooth, and predictable in ML [44, 60]. These differences between the gait of fallers and non-fallers are naturally reflected in the acceleration signal and acceleration signal frequency spectrum. Therefore, we believe that the spectral features we have proposed better reveal the changes in the spectrum.

Determining the risk of falling in the elderly in advance is a rather complicated classification problem [52]. Additionally, if the data collection is performed in a laboratory environment under direct observation, the difficulty of the classification increases furthermore, particularly in short-term recordings, since the person will walk more attentively and will not behave naturally [3]. It is known that the classification ability of gait measures obtained from accelerometers is higher compared to traditional clinical evaluations [28], and there is no relationship between gait measures and the parameters used in clinical evaluations [3]. Besides, it is unclear which feature or group of features is more predictive of a person's gait assessment [41]. Therefore, obtaining as many features as possible from acceleration signals, apart from conventional clinical parameters, may facilitate the solution to this difficult classification problem. In our study, new features not previously included in the literature have been identified, and it has been shown that they can be used to predict falls in advance. However, the result we obtained needs to be improved to reach the worldwide public population. This study has proven that success can be achieved at the multi-sensor or activity level by using the right methods, and, that a simple model can be developed with one sensor and one activity. To obtain more accurate results in future studies, experiments such as including new features, especially from the time–frequency definition, weighting the obtained features with different feature selection algorithms, and using different classification methods applicable on mobile phones are necessary.

## Conclusion

The main purpose of this study is to develop a simply applicable early fall diagnosis system based on a single sensor and a single activity. Although there are many studies in the literature conducted on the early diagnosis of falls in the elderly, a limited number are focused on such objectives. Other studies have focused on the solution through multi-sensor and/or recording during multiple activities. However, it is known that the results will not be effective in terms of clinical applicability, patient compatibility, application duration, and cost. It is clear that the problem will be solved more effectively with a single sensor and a single, simple activity. However, a single sensor and a simple activity make it difficult to solve the problem. The partially high accuracy obtained in our study shows that falling can be detected early with a sensor and a simple activity by determining the right features that reveal the structure and change of gait and can be easily applied in the assessment of the elderly during routine follow-ups in clinics. In addition, because the computational load of the features and classification model proposed in the study is applicable on mobile phones, the developed model can be integrated into mobile phone application and made available to crowds worldwide.

## Supplementary Information

Below is the link to the electronic supplementary material.

Supplementary Material 1.

## Data Availability

The datasets analyzed during the current study are publiclyavailable. 10.13026/C2S59C

## References

[1] Zhang S et al (2020) Impaired Multisensory Integration Predisposes the Elderly People to Fall: A Systematic Review. Front Neurosci 14:411. 10.3389/fnins.2020.00411 32410958 10.3389/fnins.2020.00411 PMC7198912

[2] Choi, J., et al. (2021). Wearable Sensor-Based Prediction Model of Timed up and Go Test in Older Adults. Sensors, 21(20) 10.3390/s21206831. 10.3390/s21206831 PMC8540088 34696041

[3] Bezold J et al (2021) Sensor-based fall risk assessment in older adults with or without cognitive impairment: a systematic review. Eur Rev Aging Phys Act 18(1):15. 10.1186/s11556-021-00266-w 34243722 10.1186/s11556-021-00266-w PMC8272315

[4] Demarteau J et al (2019) Trunk inclination and hip extension mobility, but not thoracic kyphosis angle, are related to 3D-accelerometry based gait alterations and increased fall-risk in older persons. Gait Posture 72:89–95. 10.1016/j.gaitpost.2019.05.027 31176286 10.1016/j.gaitpost.2019.05.027

[5] Noh B et al (2021) XGBoost based machine learning approach to predict the risk of fall in older adults using gait outcomes. Sci Rep 11(1):12183. 10.1038/s41598-021-91797-w 34108595 10.1038/s41598-021-91797-w PMC8190134

[6] Zhao, G., L. Chen, and H. Ning (2021). Sensor-Based Fall Risk Assessment: A Survey. Healthcare, 9(11) 10.3390/healthcare9111448. 10.3390/healthcare9111448 PMC8624006 34828494

[7] Rivolta MW et al (2019) Evaluation of the Tinetti score and fall risk assessment via accelerometry-based movement analysis. Artif Intell Med 95:38–47. 10.1016/j.artmed.2018.08.005 30195985 10.1016/j.artmed.2018.08.005

[8] Arganaras, J.G., et al. (2021). State-of-the-Art Wearable Sensors and Possibilities for Radar in Fall Prevention. Sensors, 21(20) 10.3390/s21206836. 10.3390/s21206836 PMC8539234 34696046

[9] Kalache, A., et al., *World Health Organisation Global Report on Falls Prevention in Older Age*, D.O.A.a.L. Course, Editor. 2008, World Health Organization: Geneva, Switzerland.

[10] Ren L, Peng Y (2019) Research of Fall Detection and Fall Prevention Technologies: A Systematic Review. IEEE Access 7:77702–77722. 10.1109/access.2019.2922708

[11] Yu L et al (2021) Assessing elderly’s functional balance and mobility via analyzing data from waist-mounted tri-axial wearable accelerometers in timed up and go tests. BMC Med Inform Decis Mak 21(1):108. 10.1186/s12911-021-01463-4 33766011 10.1186/s12911-021-01463-4 PMC7995592

[12] Buisseret, F., et al. (2020). Timed Up and Go and Six-Minute Walking Tests with Wearable Inertial Sensor: One Step Further for the Prediction of the Risk of Fall in Elderly Nursing Home People. Sensors, 20(11) 10.3390/s20113207. 10.3390/s20113207 PMC7309155 32516995

[13] Ye C et al (2020) Identification of elders at higher risk for fall with statewide electronic health records and a machine learning algorithm. Int J Med Informatics 137:104105. 10.1016/j.ijmedinf.2020.104105 10.1016/j.ijmedinf.2020.104105 32193089

[14] Bet P, Castro PC, Ponti MA (2021) Foreseeing future falls with accelerometer features in active community-dwelling older persons with no recent history of falls. Exp Gerontol 143:111139. 10.1016/j.exger.2020.111139 33189837 10.1016/j.exger.2020.111139

[15] Dubois A, Bihl T, Bresciani JP (2019) Automatic measurement of fall risk indicators in timed up and go test. Inform Health Soc Care 44(3):237–245. 10.1080/17538157.2018.1496089 30102095 10.1080/17538157.2018.1496089

[16] Zhong R, Rau PP (2020) Are cost-effective technologies feasible to measure gait in older adults? A systematic review of evidence-based literature. Arch Gerontol Geriatr 87:103970. 10.1016/j.archger.2019.103970 31743825 10.1016/j.archger.2019.103970

[17] Moncada LVV, Mire LG (2017) Preventing Falls in Older Persons. Am Fam Physician 96(4):240–247 28925664

[18] Phelan EA et al (2015) Assessment and management of fall risk in primary care settings. Med Clin North Am 99(2):281–293. 10.1016/j.mcna.2014.11.004 25700584 10.1016/j.mcna.2014.11.004 PMC4707663

[19] Najafi B et al (2002) Measurement of stand-sit and sit-stand transitions using a miniature gyroscope and its application in fall risk evaluation in the elderly. IEEE Trans Biomed Eng 49(8):843–851. 10.1109/Tbme.2002.800763 12148823 10.1109/TBME.2002.800763

[20] Howcroft, J., J. Kofman, and E.D. Lemaire (2013). Review of fall risk assessment in geriatric populations using inertial sensors. Journal of Neuroengineering and Rehabilitation, 10 10.1186/1743-0003-10-91. 10.1186/1743-0003-10-91 PMC3751184 23927446

[21] Sun TL, Huang CH (2019) Interactive visualization to assist fall-risk assessment of community-dwelling elderly people. Inf Vis 18(1):33–44. 10.1177/1473871617721243

[22] Montero-Odasso MM et al (2021) Evaluation of Clinical Practice Guidelines on Fall Prevention and Management for Older Adults: A Systematic Review. JAMA Netw Open 4(12):e2138911. 10.1001/jamanetworkopen.2021.38911 34910151 10.1001/jamanetworkopen.2021.38911 PMC8674747

[23] Yang CC, Hsu YL (2010) A review of accelerometry-based wearable motion detectors for physical activity monitoring. Sensors 10(8):7772–7788. 10.3390/s100807772 22163626 10.3390/s100807772 PMC3231187

[24] Nait Aicha, A., et al. (2018). Deep Learning to Predict Falls in Older Adults Based on Daily-Life Trunk Accelerometry. Sensors, 18(5) 10.3390/s18051654. 10.3390/s18051654 PMC5981199 29786659

[25] Bet P, Castro PC, Ponti MA (2019) Fall detection and fall risk assessment in older person using wearable sensors: A systematic review. Int J Med Inform 130:103946. 10.1016/j.ijmedinf.2019.08.006 31450081 10.1016/j.ijmedinf.2019.08.006

[26] Sun R, Sosnoff JJ (2018) Novel sensing technology in fall risk assessment in older adults: a systematic review. BMC Geriatr 18(1):14. 10.1186/s12877-018-0706-6 29338695 10.1186/s12877-018-0706-6 PMC5771008

[27] Caby B et al (2011) Feature extraction and selection for objective gait analysis and fall risk assessment by accelerometry. Biomed Eng Online 10:1–19. 10.1186/1475-925X-10-1 21244718 10.1186/1475-925X-10-1 PMC3022766

[28] Howcroft J, Lemaire ED, Kofman J (2016) Wearable-Sensor-Based Classification Models of Faller Status in Older Adults. PLoS ONE 11(4):e0153240. 10.1371/journal.pone.0153240 27054878 10.1371/journal.pone.0153240 PMC4824398

[29] Ruiz-Ruiz, L., et al. (2021). Detecting Fall Risk and Frailty in Elders with Inertial Motion Sensors: A Survey of Significant Gait Parameters. Sensors, 21(20) 10.3390/s21206918. 10.3390/s21206918 PMC8538337 34696131

[30] Sample RB et al (2017) Identification of key outcome measures when using the instrumented timed up and go and/or posturography for fall screening. Gait Posture 57:168–171. 10.1016/j.gaitpost.2017.06.007 28645093 10.1016/j.gaitpost.2017.06.007

[31] Zhou Y et al (2020) The detection of age groups by dynamic gait outcomes using machine learning approaches. Sci Rep 10(1):4426. 10.1038/s41598-020-61423-2 32157168 10.1038/s41598-020-61423-2 PMC7064519

[32] Chen SH et al (2021) Using a Stacked Autoencoder for Mobility and Fall Risk Assessment via Time-Frequency Representations of the Timed Up and Go Test. Front Physiol 12:668350. 10.3389/fphys.2021.668350 34122139 10.3389/fphys.2021.668350 PMC8194707

[33] Qiu H et al (2018) Application of Wearable Inertial Sensors and A New Test Battery for Distinguishing Retrospective Fallers from Non-fallers among Community-dwelling Older People. Sci Rep 8(1):16349. 10.1038/s41598-018-34671-6 30397282 10.1038/s41598-018-34671-6 PMC6218502

[34] Gietzelt M et al (2014) A prospective field study for sensor-based identification of fall risk in older people with dementia. Inform Health Soc Care 39(3–4):249–261. 10.3109/17538157.2014.931851 25148560 10.3109/17538157.2014.931851

[35] Greene BR et al (2014) Classification of frailty and falls history using a combination of sensor-based mobility assessments. Physiol Meas 35(10):2053–2066. 10.1088/0967-3334/35/10/2053 25237821 10.1088/0967-3334/35/10/2053

[36] Roshdibenam, V., et al. (2021). Machine Learning Prediction of Fall Risk in Older Adults Using Timed Up and Go Test Kinematics. Sensors, 21(10) 10.3390/s21103481. 10.3390/s21103481 PMC8156094 34067644

[37] Howcroft J, Kofman J, Lemaire ED (2017) Prospective Fall-Risk Prediction Models for Older Adults Based on Wearable Sensors. IEEE Trans Neural Syst Rehabil Eng 25(10):1812–1820. 10.1109/Tnsre.2017.2687100 28358689 10.1109/TNSRE.2017.2687100

[38] Iluz T et al (2015) Can a Body-Fixed Sensor Reduce Heisenberg’s Uncertainty When It Comes to the Evaluation of Mobility? Effects of Aging and Fall Risk on Transitions in Daily Living. The Journals of Gerontology: Series A 71(11):1459–1465. 10.1093/gerona/glv049 10.1093/gerona/glv049 25934996

[39] Greene BR et al (2012) Evaluation of Falls Risk in Community-Dwelling Older Adults Using Body-Worn Sensors. Gerontology 58(5):472–480. 10.1159/000337259 22571883 10.1159/000337259

[40] Marschollek, M., et al. (2011). Sensors vs. experts - a performance comparison of sensor-based fall risk assessment vs. conventional assessment in a sample of geriatric patients. BMC Med Inform Decis Mak, 11: p. 48 10.1186/1472-6947-11-48. 10.1186/1472-6947-11-48 PMC3141375 21711504

[41] Hua A et al (2018) Accelerometer-based predictive models of fall risk in older women: a pilot study. NPJ Digit Med 1:25. 10.1038/s41746-018-0033-5 31304307 10.1038/s41746-018-0033-5 PMC6550179

[42] Ihlen EAF et al (2018) Improved Prediction of Falls in Community-Dwelling Older Adults Through Phase-Dependent Entropy of Daily-Life Walking. Front Aging Neurosci 10:44. 10.3389/fnagi.2018.00044 29556188 10.3389/fnagi.2018.00044 PMC5844982

[43] Goldberger AL et al (2000) PhysioBank, PhysioToolkit, and PhysioNet: components of a new research resource for complex physiologic signals. Circulation 101(23):E215–E220. 10.1161/01.cir.101.23.e215 10851218 10.1161/01.cir.101.23.e215

[44] Weiss A et al (2013) Does the evaluation of gait quality during daily life provide insight into fall risk? A novel approach using 3-day accelerometer recordings. Neurorehabil Neural Repair 27(8):742–752. 10.1177/1545968313491004 23774124 10.1177/1545968313491004

[45] Karantonis DM et al (2006) Implementation of a real-time human movement classifier using a triaxial accelerometer for ambulatory monitoring. IEEE Trans Inf Technol Biomed 10(1):156–167. 10.1109/Titb.2005.856864 16445260 10.1109/titb.2005.856864

[46] Terrier P, Dériaz O (2011) Kinematic variability, fractal dynamics and local dynamic stability of treadmill walking. J Neuroeng Rehabil 8:12. 10.1186/1743-0003-8-12 21345241 10.1186/1743-0003-8-12 PMC3060113

[47] Antonsson EK, Mann RW (1985) The frequency content of gait. J Biomech 18(1):39–47. 10.1016/0021-9290(85)90043-0 3980487 10.1016/0021-9290(85)90043-0

[48] Dasgupta P et al (2021) Acceleration Gait Measures as Proxies for Motor Skill of Walking: A Narrative Review. IEEE Trans Neural Syst Rehabil Eng 29:249–261. 10.1109/TNSRE.2020.3044260 33315570 10.1109/TNSRE.2020.3044260 PMC7995554

[49] Zijlstra, W. (2004). Assessment of spatio-temporal parameters during unconstrained walking. Eur J Appl Physiol, 92(1–2): p. 39–44 https://www.ncbi.nlm.nih.gov/pubmed/14985994. 10.1007/s00421-004-1041-5 14985994

[50] Kononenko I, Šimec E, Robnik-Šikonja M (1997) Overcoming the Myopia of Inductive Learning Algorithms with RELIEFF. Appl Intell 7(1):39–55. 10.1023/A:1008280620621

[51] Robnik-Sikonja, M. and I. Kononenko. *An adaptation of Relief for attribute estimation in regression*. in *Proceedings of the Fourteenth International Conference on Machine Learning*. 1997. San Francisco (United States): Morgan Kaufmann Publishers Inc.

[52] Howcroft, J., J. Kofman, and E.D. Lemaire (2017). Feature selection for elderly faller classification based on wearable sensors. Journal of Neuroengineering and Rehabilitation, 14 10.1186/s12984-017-0255-9. 10.1186/s12984-017-0255-9 PMC5450084 28558724

[53] Svozil D, Kvasnicka V, J.í. Pospichal, (1997) Introduction to multi-layer feed-forward neural networks. Chemom Intell Lab Syst 39(1):43–62. 10.1016/S0169-7439(97)00061-0

[54] Haykin S (2001) Neural Networks: A Comprehensive Foundation, 2nd edn. Prentice Hall PTR, India

[55] Demšar J et al (2013) Orange: Data Mining Toolbox in Python. J Mach Learn Res 14:2349–2353

[56] Pedregosa F et al (2011) Scikit-learn: Machine Learning in Python. J Mach Learn Res 12:2825–2830

[57] Shany, T., et al. (2012). Assessing fall risk using wearable sensors: a practical discussion. A review of the practicalities and challenges associated with the use of wearable sensors for quantification of fall risk in older people. Z Gerontol Geriatr, 45(8): p. 694–706 10.1007/s00391-012-0407-2. 10.1007/s00391-012-0407-2 23184295

[58] Bautmans I et al (2011) Reliability and clinical correlates of 3D-accelerometry based gait analysis outcomes according to age and fall-risk. Gait Posture 33(3):366–372. 10.1016/j.gaitpost.2010.12.003 21227697 10.1016/j.gaitpost.2010.12.003

[59] Gupta P, Dallas T (2014) Feature Selection and Activity Recognition System Using a Single Triaxial Accelerometer. IEEE Trans Biomed Eng 61(6):1780–1786. 10.1109/TBME.2014.2307069 24691526 10.1109/TBME.2014.2307069

[60] van Schooten KS et al (2016) Daily-Life Gait Quality as Predictor of Falls in Older People: A 1-Year Prospective Cohort Study. PLoS ONE 11(7):e0158623. 10.1371/journal.pone.0158623 27389583 10.1371/journal.pone.0158623 PMC4936679

[61] Marschollek M et al (2011) Sensor-based Fall Risk Assessment – an Expert ‘to go.’ Methods Inf Med 50(05):420–426. 10.3414/ME10-01-0040 21206963 10.3414/ME10-01-0040

[62] Montesinos L, Castaldo R, Pecchia L (2018) Wearable Inertial Sensors for Fall Risk Assessment and Prediction in Older Adults: A Systematic Review and Meta-Analysis. IEEE Trans Neural Syst Rehabil Eng 26(3):573–582. 10.1109/TNSRE.2017.2771383 29522401 10.1109/TNSRE.2017.2771383

[63] Cho C-Y, Kamen G (1998) Detecting Balance Deficits in Frequent Fallers Using Clinical and Quantitative Evaluation Tools. J Am Geriatr Soc 46(4):426–430. 10.1111/j.1532-5415.1998.tb02461.x 9560063 10.1111/j.1532-5415.1998.tb02461.x

