# Supplementary material for: Leveraging feature selection for enhanced fall risk prediction in elderly using gait analysis
Source: Med Biol Eng Comput. 2024 Aug 10;62(12):3887–97. doi: 10.1007/s11517-024-03180-2 (PMC11568989; doi:10.1007/s11517-024-03180-2)
Supplement: Supplementary file 1 — Supplementary Material 1. [file 11517_2024_3180_MOESM1_ESM.docx]

**Acceleration gait measures**

A detailed description of the acceleration gait measures is given below. The 53 features between the 10th and 62nd have been calculated separately for each acceleration axis, resulting in a total of 168 features. The axis of the relevant measurement is given in the suffix (ML, AP, V) added to the measure names in manuscript.

1. Average Step Duration (AvStpDur): Between two consecutive local minimum points in the AP axis acceleration signal shows the step duration [1]. The average of the durations between local minimum points found for the entire axis is considered the average step duration [2].
2. Average Stride Duration (AvStrDur): The average of two consecutive steps duration [2].
3. Cadence (Cadence): The walking speed of a person is referenced as the number of steps per minute.
4. Correlation between vertical and mediolateral axes acceleration (corr_V_ML): The correlation between acceleration signals recorded from vertical and mediolateral axes.
5. Correlation between vertical and anterior-posterior axes acceleration (corr_V_AP): The correlation between acceleration signals recorded from vertical and anterior-posterior axes.
6. Correlation between mediolateral and anterior-posterior axes acceleration (corr_ML_AP): The correlation between acceleration signals recorded from mediolateral and anterior-posterior axes.
7. Covariance between vertical and anterior-posterior axes acceleration (cov_V_ML): The correlation between acceleration signals recorded from vertical and mediolateral axes.
8. Covariance between vertical and mediolateral axes acceleration (cov_V_AP): The correlation between acceleration signals recorded from vertical and anterior-posterior axes.
9. Covariance between mediolateral and anterior-posterior axes acceleration (cov_ML_AP): The correlation between acceleration signals recorded from mediolateral and anterior-posterior axes.
10. Step Regularity (StpReg): The correlation between the acceleration signal and the new signal obtained by shifting the acceleration signal by the average step duration [3].
11. Stride Regularity (StrReg): The correlation between the acceleration signal and the signal obtained by shifting the acceleration signal by the average stride duration [3].
12. Step Symmetry (StpSym): The percentage difference between step regularity and stride regularity [3].

$$\mathrm{StpSym}=\frac{\left| \mathrm{StpReg}-\mathrm{StrReg} \right|}{\left( \mathrm{StpReg}+\mathrm{StrReg} \right)/2}.100$$

13. Harmonic Ratio (HR): The harmonic ratio is a measure of the smoothness of walking, and it is defined as the ratio of the amplitude sum of the even harmonics of the acceleration signal to the amplitude sum of the odd harmonics of the acceleration signal [4].
14. Mean (Mean): The average of the acceleration signal amplitude [5].
15. First Half Mean (MeanFH): The average of the acceleration signal amplitude from the beginning of the step to the middle of the step [6].
16. Last Half Mean (MeanLH): The average of the acceleration signal amplitude from the middle of the step to the end of the step [6].
17. Standard Deviation (sd): The standard deviation of the acceleration signal amplitude [5].
18. Median (Med): The median of the acceleration signal amplitude [7].
19. Variation (Var): The variance of the acceleration signal amplitude.
20. Skewness (Skew): It characterizes the degree of asymmetry of the distribution around the mean [5] and it is calculated by

$$Skew=\frac{E\left( x-\mu\right)^{3}}{\sigma^{3}}$$

where *x* refers to the acceleration signal, *μ* refers to the acceleration signal average, σ refers to the acceleration signal standard deviation, and *E* refers to the expected value.

1. Kurtosis (Kurt): It characterizes the degree of steepness and flatness of the distribution around the mean [5].

$$Kurt=\frac{E\left( x-\mu\right)^{4}}{\sigma^{4}}$$

2. Interquartile Range (iqr): The difference between the third quartile and the first quartile range of the acceleration signal amplitude [5].
3. Average Absolute Difference (Av_Ab_Diff): The average of the differences between each value of the acceleration signal amplitude and its arithmetic mean [8].
4. Root Mean Square (rms): The effective value of the acceleration signal [9].
5. Signal Magnitude Area (sma): The area below the averaged acceleration signal [10].
6. Number of Peaks (Npeaks): The number of peaks in the acceleration signal [11].
7. Mean of Peaks Amplitude (meanPeaks): The average of the peak values in the acceleration signal.
8. Shanon Entropy (ShanEnp): Shannon entropy is a measure of the uncertainty of a random process [12]. It is calculated by

$$\mathrm{ShanEnp}\left( x \right)=-\sum_{i} {x(i)}^{2}\log({x(i)}^{2})$$

where *x* refers to the acceleration signal, *i* refers to the time index.

1. Dynamic Time Warping Distance (dtwd): Dynamic time warping is an algorithm that calculates the similarity of time series signals [13]. In this study, it is used as a measure of the similarity of the signals of consecutive steps. It is calculated by

$$dtwd=\sqrt{\sum_{i=1}^{N} \left( x(i)-y(i) \right)^{2}}$$

where x is the acceleration signal, *y* is the acceleration signal delayed by one step, *i* is the time index.

1. Mean Trend (MeanTrend) The acceleration signal first is divided into half-second windows and the acceleration signal is averaged for each window. The sum of the absolute differences of the averages of consecutive windows provides the mean trend [14]. The mean trend is calculated by

$$MeanTrend=\sum\left( \left| \mu_{i}-\mu_{i-1} \right| \right)$$

where µ_i_ is average of the ith window.

1. Dominant Frequency (F1): Frequency at which the PSD of the acceleration signal is maximum [11].
2. Second Dominant Frequency (F2): Frequency at which the PSD of the acceleration signal is the second maximum [11].
3. Amplitude of Dominant Frequency (AmpF1): Amplitude of F1.
4. Amplitude of Second Dominant Frequency (AmpF2): Amplitude of F2.
5. Width of Dominant Frequency (wF1): The measure of the steepness of the F1 peak, indicating the frequency difference between points on both sides of the F1 peak where amplitude decreases to half of AmpF1 [15].
6. Width of Second Dominant Frequency (wF2): The measure of the steepness of the F2 peak, indicating the frequency difference between points on both sides of the F2 peak where amplitude decreases to half of AmpF2 [15].
7. Prominence of Dominant Peak (pF1): Relative height of first dominant peak [11].
8. Prominence of Second Dominant Peak (pF2): Relative height of second dominant peak [11].
9. Occupied Bandwidth (obw): Integrated power crossing the rate of 0.5% and 99.5% of the overall power in the frequency spectrum based on the difference in frequency between the points.
10. Mean of Peaks (pksMean): Mean of all peak values of the frequency spectrum.
11. Absolute Difference between F1 and F2 (diff_F1F2): Absolute differences of F1 and F2.
12. Quality Factor1 (QF1): Quality factor of F1 (QF=F1/wF2).
13. Quality Factor2 (QF2): Quality factor of F2 (QF=F2/wF2).
14. Area Under the PSD (Area_PSD): Area under all spectra.
15. Area Under the PSD before First Peak (Area_BF1): Area under the frequency spectrum between zero to F1 band.
16. Area Under the PSD after First Peak (Area_AF1): Area under the frequency spectrum between F1 to infinity band.
17. Ratio of Area_BF1 to Area_AF1 (Area_R): Ratio of Area_BF1 to Area_AF1 (Area_AF1/Area_BF1).
18. Mean of the PSD (Mean_PSD): Mean of PSD.
19. Mean of the First Half of PSD (Mean_FH_PSD): Mean of PSD from 0 Hz to 7 Hz band
20. Mean of the Last Half of PSD (Mean_LH_PSD): Mean of PSD from 7 Hz to 15 Hz band
21. Spectral Median (medPSD): Median of PSD of the acceleration signal.
22. Spectral Variance (varPSD): Variance of PSD of the acceleration signal.
23. Interquartile range of PSD (iqrPSD): It is the difference between the third quartile and the first quartile range of PSD of the acceleration signal.
24. Square Root Mean of PSD (rmsPSD): It is the effective value of PSD of the acceleration signal.
25. Mean Absolute Deviation of PSD (madPSD): It is the average of the differences between each value of the acceleration signal and the arithmetic mean.
26. Spectral Centroid (centroid_PSD): It indicates the location of the center of the frequency spectrum's mass[16]. It is calculated based on

$$\mathrm{centroid}_{\mathrm{PSD}}=\frac{\sum_{k} \left[ X\left( k \right).f\left( k \right) \right]}{\sum_{k} X\left( k \right)}$$

where *X* is the frequency spectrum, *f* is the frequency, and *k* is the frequency index.

1. Spectral Spread (spread_PSD): The spread of the spectrum around its mean value [16]. It is calculated based on

$$\text{spread\_PSD}=\sqrt{\frac{\sum_{k} \left( f\left( k \right)-centroid\_PSD \right)^{2}X(k)}{\sum_{k} X(k)}}$$

2. Spectral Skewness (skew_PSD): It characterizes the degree of asymmetry of the distribution around the mean [16]. It is calculated based on

$$\text{skew\_PSD}=\frac{\sum_{k} \left( f\left( k \right)-centroid_{\mathrm{PSD}} \right)^{3}X(k)}{\left( spread\_PSD \right)^{3}\sum_{k} X(k)}$$

3. Spectral Kurtosis (kurt_PSD): It characterizes the degree of steepness and flatness of the distribution around the mean [16]. It is calculated based on

$$\text{kurt\_PSD}=\frac{\sum_{k} \left( f\left( k \right)-centroid_{\mathrm{PSD}} \right)^{4}X(k)}{\left( spread\_PSD \right)^{4}\sum_{k} X(k)}$$

4. Spectral Decrease (decrease_PSD): It represents the amount of decreasing of the spectral amplitude [16]. It is calculated based on

$$decrease\_PSD=\frac{1}{\sum_{k=2:K} X(k)}\sum_{k=2:K} \frac{X\left( k \right)-X(1)}{k-1}$$

5. Spectral Flatness (flatness_PSD) It is a measure of the flatness of frequency spectrum [16]. It is calculated based on

$$flatness\_PSD=\frac{\left[ \prod_{k} X(k) \right]^{\frac{1}{K}}}{\frac{1}{K}\sum_{k} X(k)}$$

6. Spectral Crest (crest_PSD): It is another descriptor for the flatness of the frequency spectrum. It is calculated by the ratio of the maximum value of the frequency spectrum to the arithmetic mean of the frequency spectrum [16]. It is calculated based on

$$crest\_PSD=\frac{max(X(k))}{\frac{1}{K}\sum_{k} X(k)}$$

**REFERENCES**

1. Terrier, P. and O. Dériaz (2011). Kinematic variability, fractal dynamics and local dynamic stability of treadmill walking. J Neuroeng Rehabil, 8: p. 12 https://doi.org/10.1186/1743-0003-8-12.

2. Barden, J.M., et al. (2016). Accelerometer-Based Step Regularity Is Lower in Older Adults with Bilateral Knee Osteoarthritis. Frontiers in Human Neuroscience, 10 https://doi.org/10.3389/fnhum.2016.00625.

3. Kobsar, D., et al. (2014). Evaluation of age-related differences in the stride-to-stride fluctuations, regularity and symmetry of gait using a waist-mounted tri-axial accelerometer. Gait & Posture, 39(1): p. 553-557 https://doi.org/10.1016/j.gaitpost.2013.09.008.

4. Bellanca, J.L., et al. (2013). Harmonic ratios: A quantification of step to step symmetry. Journal of Biomechanics, 46(4): p. 828-831 https://doi.org/10.1016/j.jbiomech.2012.12.008.

5. Guo, C., M. Lu, and J. Chen (2020). An evaluation of time series summary statistics as features for clinical prediction tasks. BMC Medical Informatics and Decision Making, 20(1): p. 48 https://doi.org/10.1186/s12911-020-1063-x.

6. Pires, I.M., et al. (2020). Pattern Recognition Techniques for the Identification of Activities of Daily Living Using a Mobile Device Accelerometer. Electronics, 9(3): p. 509 https://doi.org/10.3390/electronics9030509.

7. Wannenburg, J. and R. Malekian (2017). Physical Activity Recognition From Smartphone Accelerometer Data for User Context Awareness Sensing. IEEE Transactions on Systems, Man, and Cybernetics: Systems, 47(12): p. 3142-3149 https://doi.org/10.1109/TSMC.2016.2562509.

8. Kwapisz, J.R., G.M. Weiss, and S.A. Moore (2011). Activity recognition using cell phone accelerometers. SIGKDD Explor. Newsl., 12(2): p. 74–82 https://doi.org/10.1145/1964897.1964918.

9. Menz, H.B., S.R. Lord, and R.C. Fitzpatrick (2003). Acceleration patterns of the head and pelvis when walking on level and irregular surfaces. Gait & Posture, 18(1): p. 35-46 https://doi.org/10.1016/S0966-6362(02)00159-5.

10. Lavanya, B. and G.S. Gayathri. *Exploration and Deduction of Sensor-Based Human Activity Recognition System of Smart-Phone Data*. in *2017 IEEE International Conference on Computational Intelligence and Computing Research (ICCIC)*. 2017.

11. Marschollek, M., et al. (2011). Sensor-based Fall Risk Assessment – an Expert ‘to go’. Methods Inf Med, 50(05): p. 420-426 https://doi.org/10.3414/ME10-01-0040.

12. Diego Galar, U.K., *eMaintenance: Essential Electronic Tools for Efficiency*. Chapter 3 - Preprocessing and Features. 2017: Academic Press.

13. Sakoe, H. and S. Chiba (1978). Dynamic programming algorithm optimization for spoken word recognition. IEEE Transactions on Acoustics, Speech, and Signal Processing, 26(1): p. 43-49 https://doi.org/10.1109/TASSP.1978.1163055.

14. Gupta, P. and T. Dallas (2014). Feature Selection and Activity Recognition System Using a Single Triaxial Accelerometer. Ieee Transactions on Biomedical Engineering, 61(6): p. 1780-1786 https://doi.org/10.1109/TBME.2014.2307069.

15. Weiss, A., et al. (2011). Toward Automated, At-Home Assessment of Mobility Among Patients With Parkinson Disease, Using a Body-Worn Accelerometer. Neurorehabilitation and Neural Repair, 25(9): p. 810-818 https://doi.org/10.1177/1545968311424869.

16. Peeters, G., *A Large Set of Audio Features for Sound Description (Similarity and Classification) in the CUIDADO Project.* 2004, Institut de Recherche et de Coordination Acoustique (IRCAM): Paris, France.
